# Supplementary material for: Caffeine-inducible gene switches controlling experimental diabetes
Source: Nat Commun. 2018 Jun 19;9:2318. doi: 10.1038/s41467-018-04744-1 (PMC6008335; doi:10.1038/s41467-018-04744-1)
Supplement: Supplementary file 1 — Supplementary Information [file 41467_2018_4744_MOESM1_ESM.pdf]

Supplementary Information

Caffeine-inducible gene switches controlling experimental diabetes

Daniel Bojar, Leo Scheller, Ghislaine Charpin-El Hamri, Mingqi Xie, Martin Fussenegger

Supplementary Figures

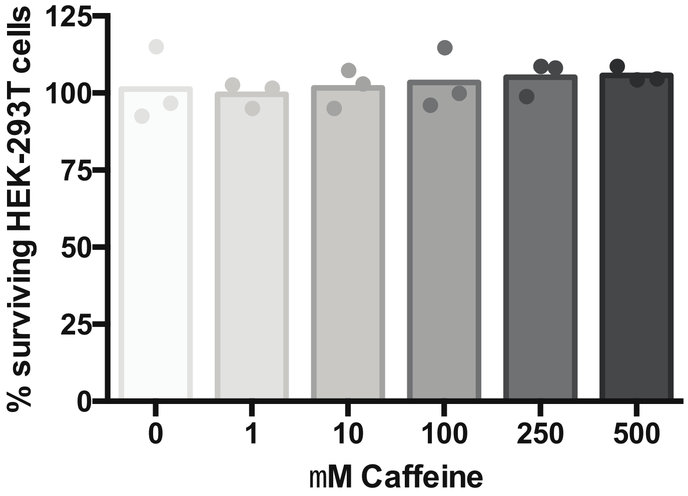

Supplementary Figure 1 | Viability of HEK-293T cells in the presence of caffeine.

HEK-293T cells were exposed to increasing concentrations of caffeine in standard cell culture medium. After 24 hours, cellular viability was assessed with a CCK-8 assay. Data are shown as the mean in bar graphs and symbols indicate individual data points. The data displayed represent three independent experiments (n = 3).

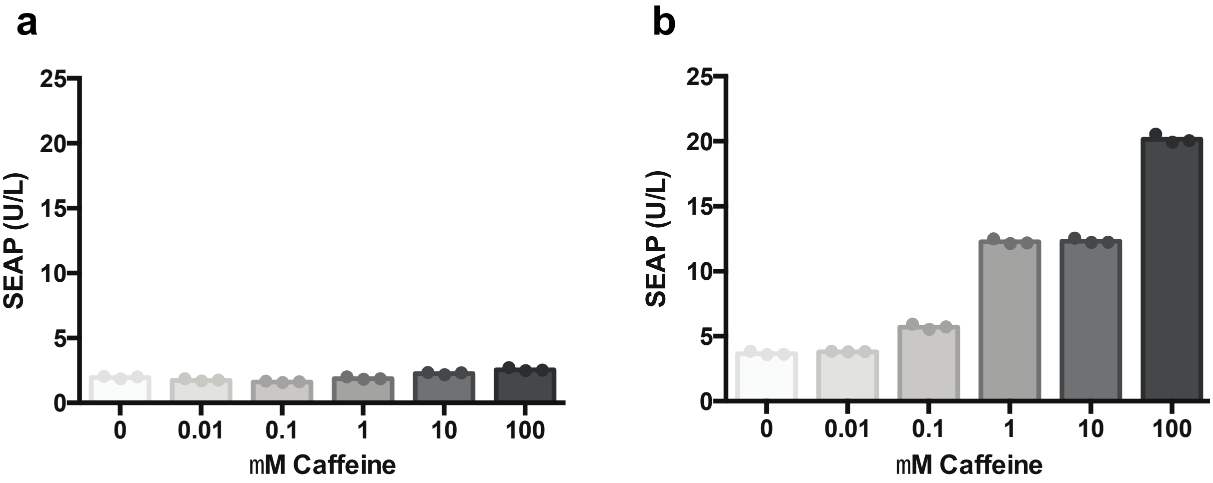

Supplementary Figure 2 | Combination of STAT3- and TetR-signaling by caffeine leads to nonlinearity.

a) Orthogonality of STAT3 signaling and TetR-dependent promoter. HEK-293T cells transfected with pDB306 (P_{hCMV}-aCaffVHH-EpoR$_m$-IL-6RB$_m$-pA$_{bGH}$) and pMF111 (P_{tetO7}-SEAP-pA$_{SV40}$) were exposed

to increasing concentrations of caffeine in standard cell culture medium 16 hours after transfection. SEAP was measured 24 hours after the addition of caffeine in the supernatant of the cells. **b)** Nonlinear response with the combination of caffeine-sensing systems. HEK-293T cells transfected with pDB306 (P_{hCMV}-aCaffVHH-EpoR$_m$-IL-6RB$_m$-pA$_{bGH}$), pLS13 (P_{STAT3}-SEAP-pA$_{SV40}$), pDB307 (P_{SV40}-TetR-aCaffVHH-pA$_{SV40}$), pDB335 (P_{CAG}-aCaffVHH-VP$_{minx4}$-pA$_{\beta G}$) and pMF111 (P_{tetO7}-SEAP-pA$_{SV40}$) were exposed to increasing concentrations of caffeine in standard cell culture medium 16 hours after transfection. SEAP was measured 24 hours after the addition of caffeine in the supernatant of the cells. Data are shown as the mean in bar graphs and symbols indicate individual data points. The data displayed represent three independent experiments (n = 3).

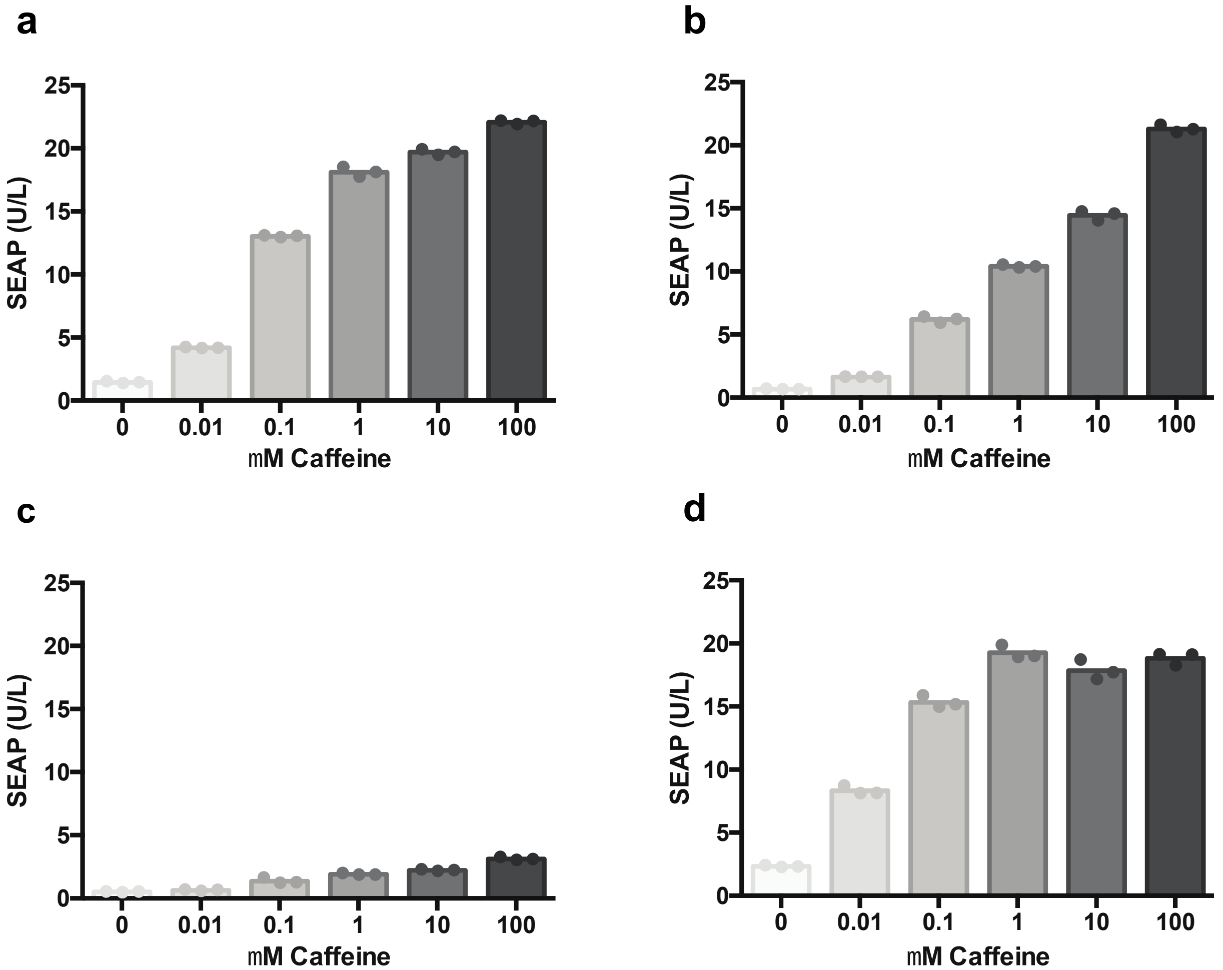

Supplementary Figure 3 | Caffeine sensitivity of monoclonal C-STAR cell lines. (**a**) C-STAR$_{DB2}$, (**b**) C-STAR$_{DB3}$, (**c**) C-STAR$_{DB4}$ and (**d**) C-STAR$_{DB5}$ cells transfected with the reporter plasmid pLS13 (P_{STAT3}-SEAP-pA$_{SV40}$) were exposed to increasing concentrations of caffeine in standard cell culture medium at 16 hours after transfection. SEAP was measured 24 hours after the addition of caffeine in the supernatant of the cells. Data are shown as the mean in bar graphs and symbols indicate individual data points. The data displayed represent three independent experiments (n = 3).

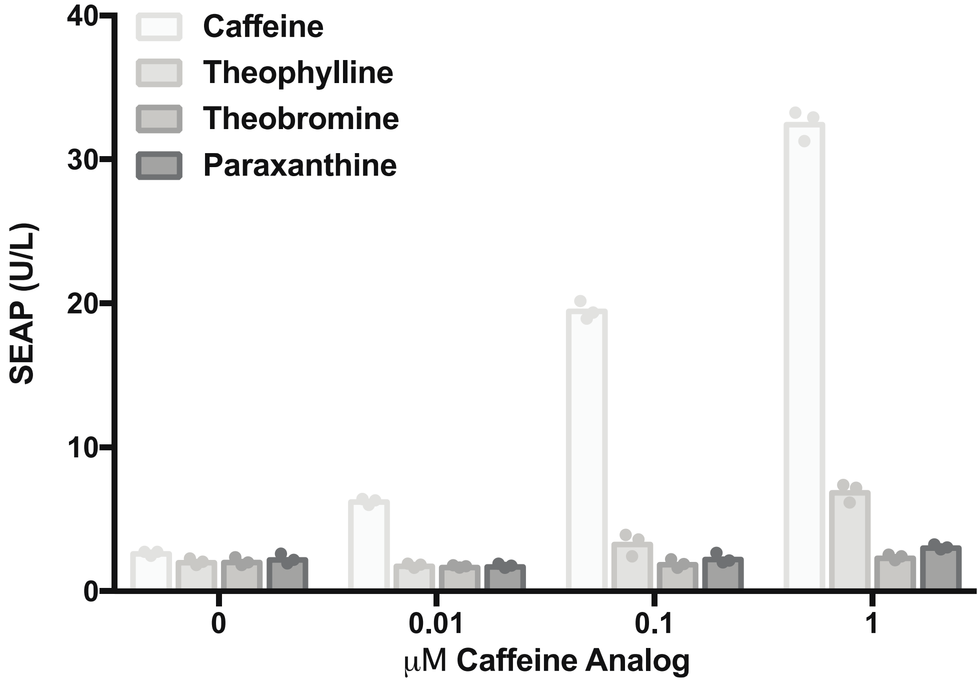

Supplementary Figure 4 | Specificity of C-STAR$_{DB1}$ for caffeine versus several analogs. C-STAR$_{DB1}$ cells transfected with the reporter plasmid pLS13 (P$_{STAT3}$-SEAP-pA$_{SV40}$) were exposed to increasing concentrations of caffeine, theophylline, theobromine and paraxanthine in standard cell culture medium at 16 hours after transfection. SEAP was measured 24 hours after the addition of caffeine in the supernatant of the cells. Data are shown as the mean in bar graphs and symbols indicate individual data points. The data displayed represent three independent experiments (n = 3).

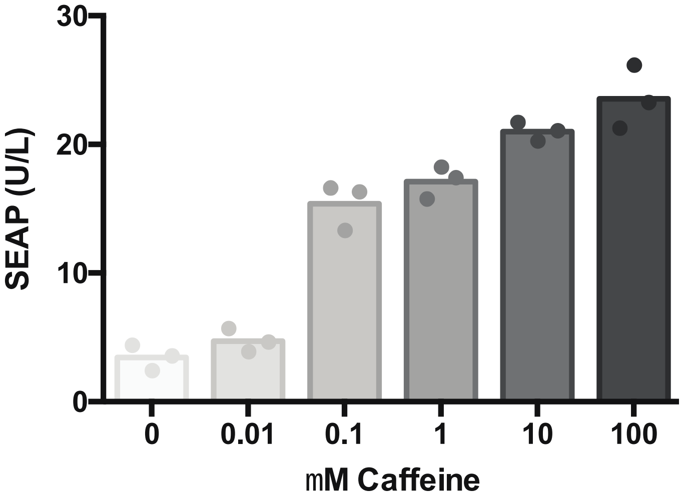

Supplementary Figure 5 | Functionality of encapsulated C-STAR$_{DB1}$ cells in the presence of caffeine. C-STAR$_{DB1}$ cells transfected with the reporter plasmid pLS13 (P$_{STAT3}$-SEAP-pA$_{SV40}$) and encapsulated in vascularized microcontainers were exposed to increasing concentrations of caffeine in standard cell culture medium. After 24 hours, SEAP activity was quantified in the supernatant of the cells. Data are shown as the mean in bar graphs and symbols indicate individual data points. The data displayed represent three independent experiments (n = 3).

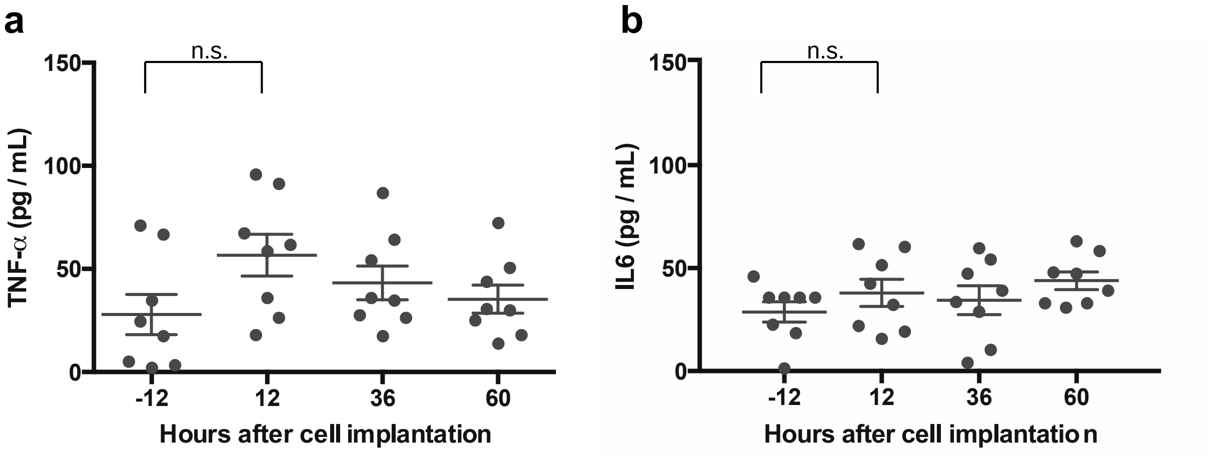

Supplementary Figure 6 | Immunogenic impact of microcapsule implants. Wild-type mice were intraperitoneally implanted with microencapsulated C-STAR$_{DB6}$ cells and blood samples were collected at 12 hours before (control) and at 12, 36 and 60 hours after implantation. **(a)** TNF-α and **(b)** IL-6 levels in the bloodstream of mice were determined by ELISA. The data displayed are mean ± SEM (n = 8 mice). n.s. not significant (Welch's t test).

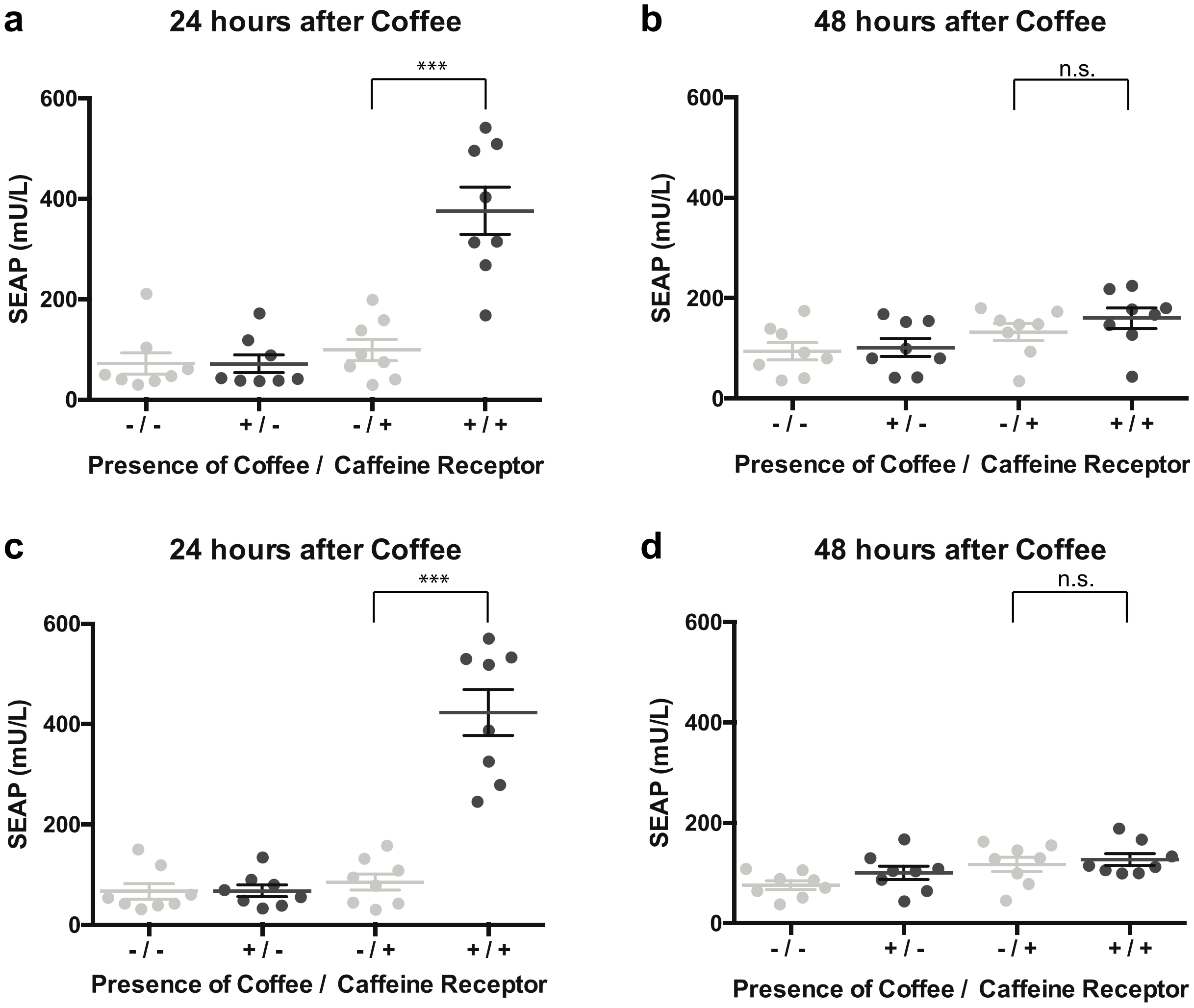

Supplementary Figure 7 | Reversible stimulation of C-STAR$_{DB1}$ *in vivo*. Wild-type mice were intraperitoneally implanted with microencapsulated C-STAR$_{DB1}$ (Caffeine Receptor: +) or control HEK-293T cells only transfected with pLS13 (Caffeine Receptor: -; P_{STAT3}-SEAP-pA_{SV40}), and stimulated by oral administration of 300 µL Volluto® coffee (Coffee: +) or H_2O (Coffee: -). **(a-b)** SEAP levels in the bloodstream of mice were quantified at **(a)** 24 h and **(b)** 48 h after coffee intake. **(c-d)** Five days after implantation, the same mice as described in **(a-b)** received another oral administration of 300 µL Volluto® coffee (Coffee: +) or H_2O (Coffee: -). SEAP levels in the bloodstream were quantified at **(c)** 24 h and **(d)** 48 h after the second coffee intake. The data displayed are mean ± SEM (n = 8). ***P < 0.001 versus control, n.s. not significant (Welch's t test).

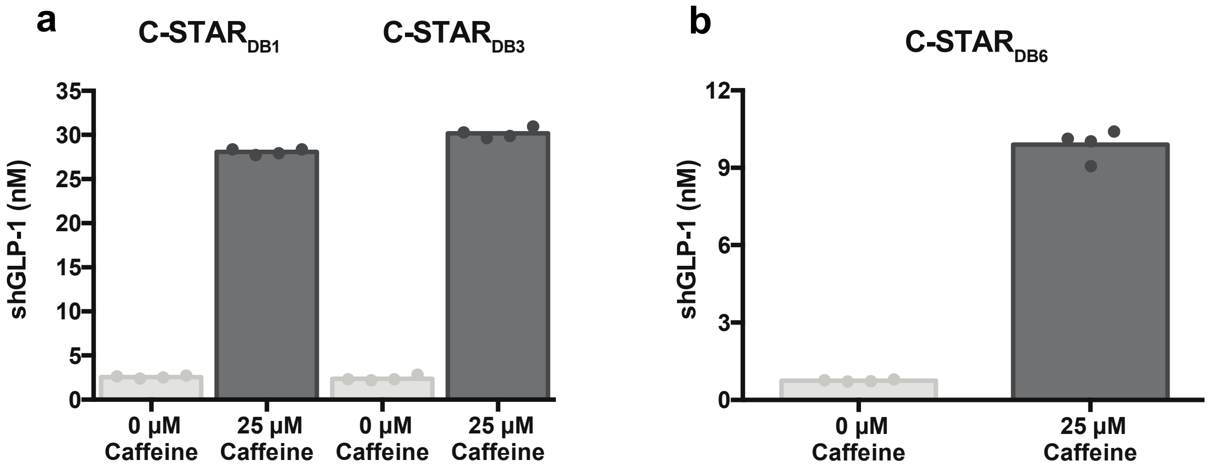

Supplementary Figure 8 | Caffeine-dependent shGLP-1 expression *in vitro*.

a) Assessment of the caffeine-induced expression of shGLP-1. C-STAR$_{DB1}$ or C-STAR$_{DB3}$ cells stably expressing the caffeine receptor ($P_{hEF-1\alpha}$-aCaffVHH-EpoR$_m$-IL-6RB$_m$-pA$_{SV40}$, pDB326) were transiently transfected with pDB387 (P_{STAT3}-shGLP-1-pA$_{SV40}$) and exposed to H_2O or caffeine 16 hours after transfection. After 48 hours, shGLP-1 expression was quantified with a Mouse IgG ELISA Kit (ICL Lab) in the supernatant of the cells. The data displayed represent four independent experiments (n = 4). **b)** Validation of the polyclonal, shGLP-1 expressing C-STAR$_{DB6}$ cell line. C-STAR$_{DB6}$ cells stably expressing both the caffeine receptor ($P_{hEF-1\alpha}$-aCaffVHH-EpoR$_m$-IL-6RB$_m$-pA$_{SV40}$, pDB326) as well as shGLP1 (P_{STAT3}-shGLP-1-pA$_{SV40}$, pDB387) were exposed to H_2O or caffeine. After 48 hours, shGLP-1 expression was quantified with a Mouse IgG ELISA Kit (ICL Lab) in the supernatant of the cells. Data are shown as the mean in bar graphs and symbols indicate individual data points. The data displayed represent four independent experiments (n = 4).

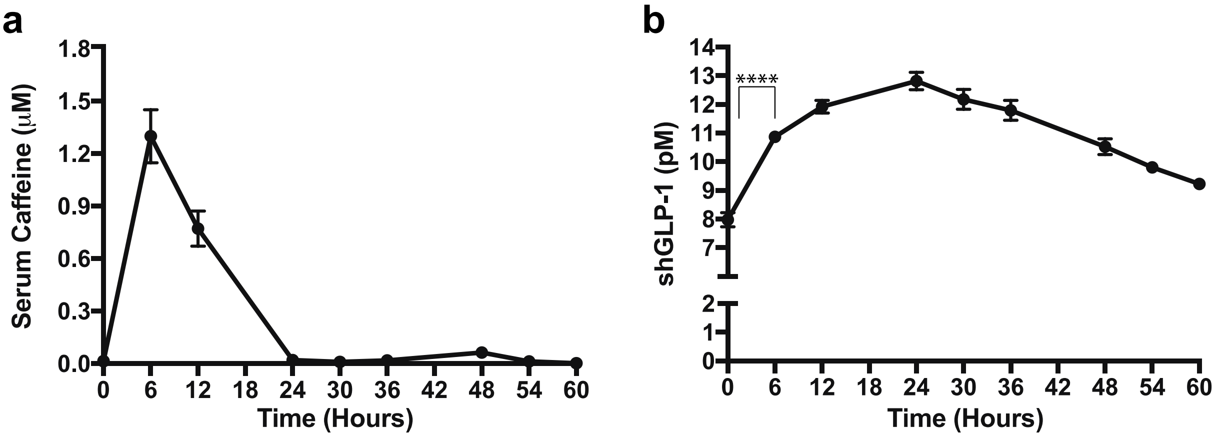

Supplementary Figure 9 | Pharmacokinetics of caffeine and shGLP-1 *in vivo*. Wild-type mice were intraperitoneally implanted with microencapsulated C-STAR$_{DB6}$ cells and received a single oral administration of 300 μL Nespresso Volluto® coffee. **(a)** Caffeine and **(b)** shGLP-1 levels in the bloodstream of mice were recorded every six hours for 60 hours. The data displayed are mean ± SEM (n = 8). ****P < 0.0001 versus control (Welch's t test).

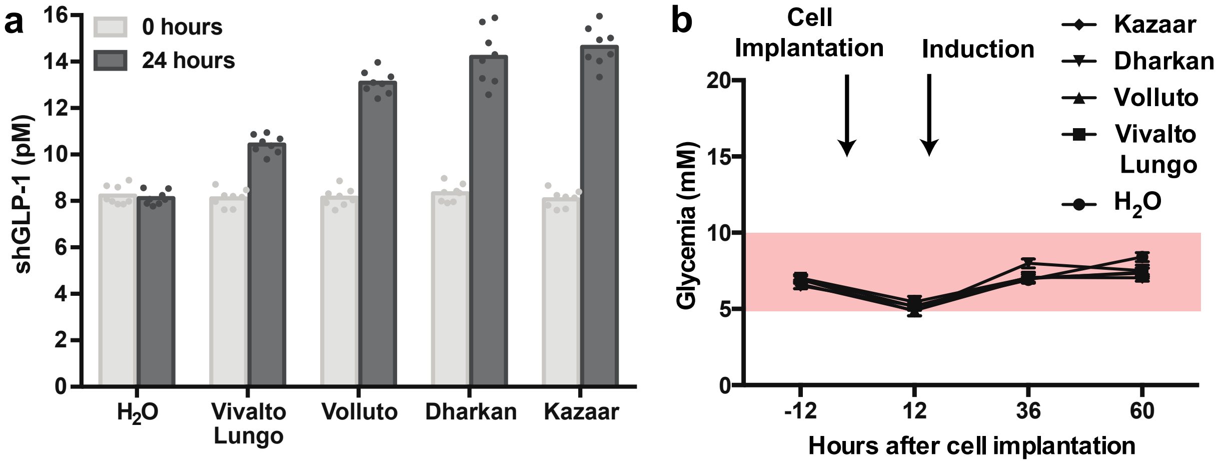

Supplementary Figure 10 | Coffee dose-dependence *in vivo*. (a,b) Wild-type mice were intraperitoneally implanted with microencapsulated C-STAR$_{DB6}$ cells, and received single oral administrations of different Nespresso® coffee formulations having different caffeine concentrations (ingestion volume: 300 µL). **(a)** Dose-dependent production of shGLP-1 in the bloodstream of mice was recorded 24 h after coffee intake. Data are shown as the mean in bar graphs and symbols indicate individual mice (n = 8). **(b)** Fasting glycemia was recorded for 72 h. The range of homeostatic fasting glycemia is indicated with a red box. The data displayed are mean ± SEM (n = 8 mice). Welch's t test showed no significant differences between groups.
