# Supplementary material for: Caffeine-inducible gene switches controlling experimental diabetes
Source: Nat Commun. 2018 Jun 19;9:2318. doi: 10.1038/s41467-018-04744-1 (PMC6008335; doi:10.1038/s41467-018-04744-1)
Supplement: Supplementary file 3 — Description of Additional Supplementary Files [file 41467_2018_4744_MOESM3_ESM.pdf]

Description of Additional Supplementary Files

File Name: Supplementary Data 1
Description: Plasmids used and designed in this study.
